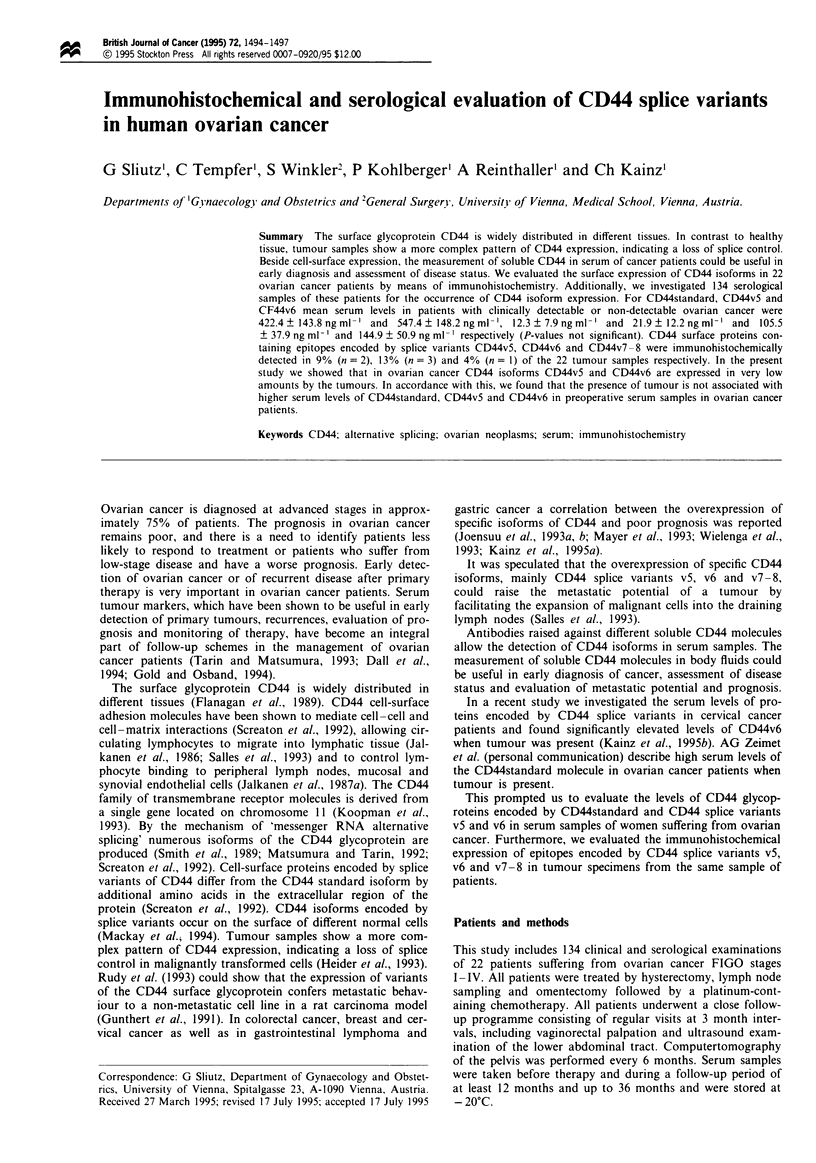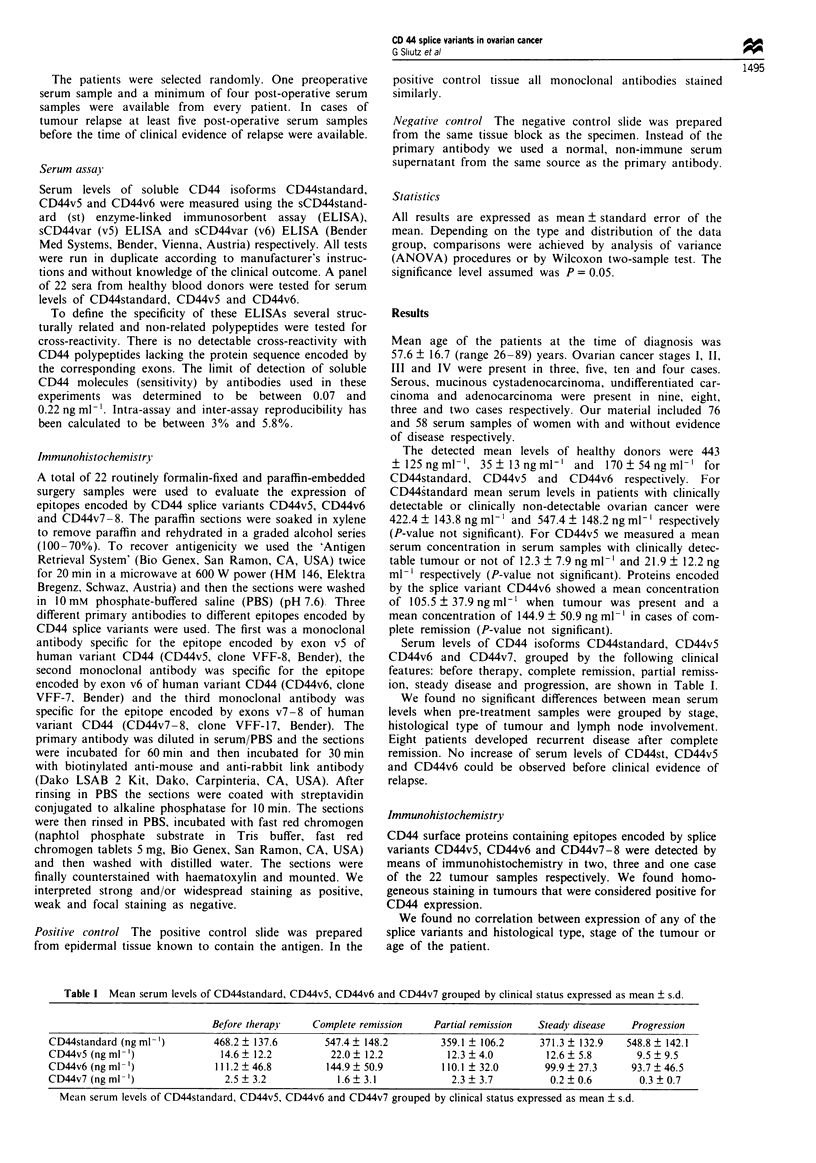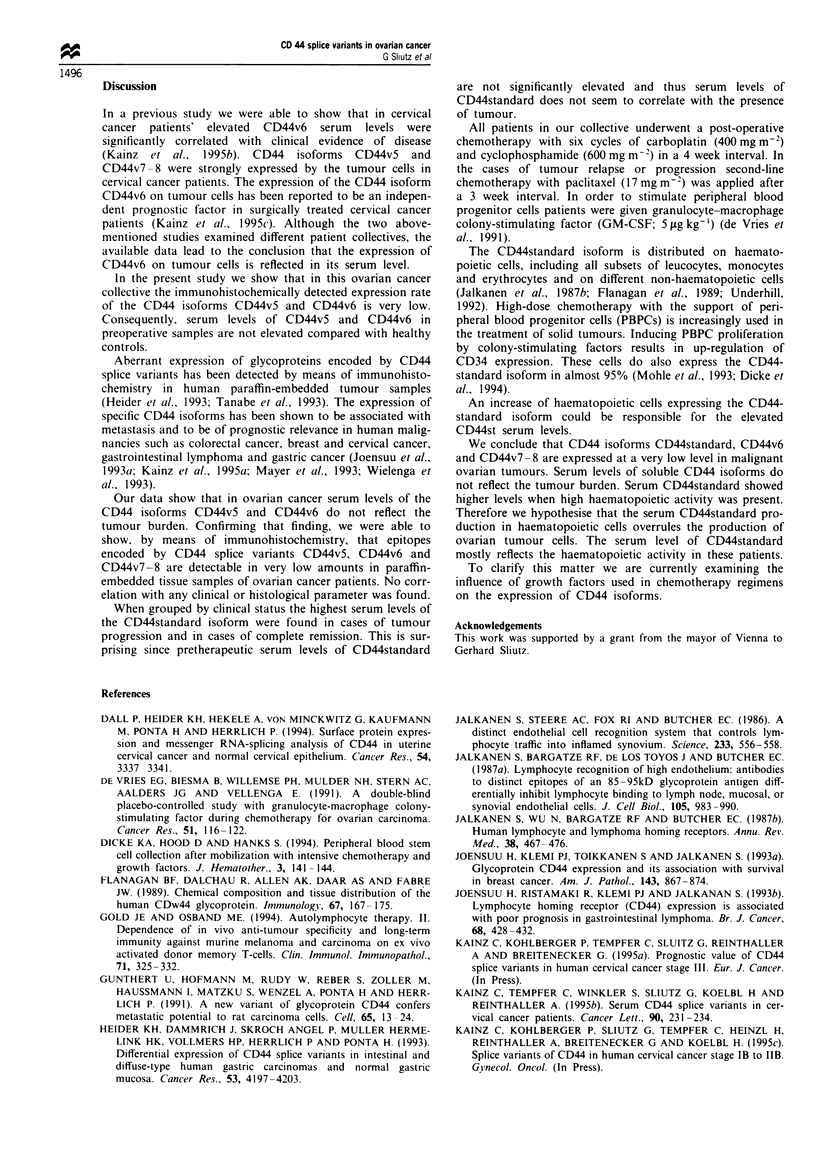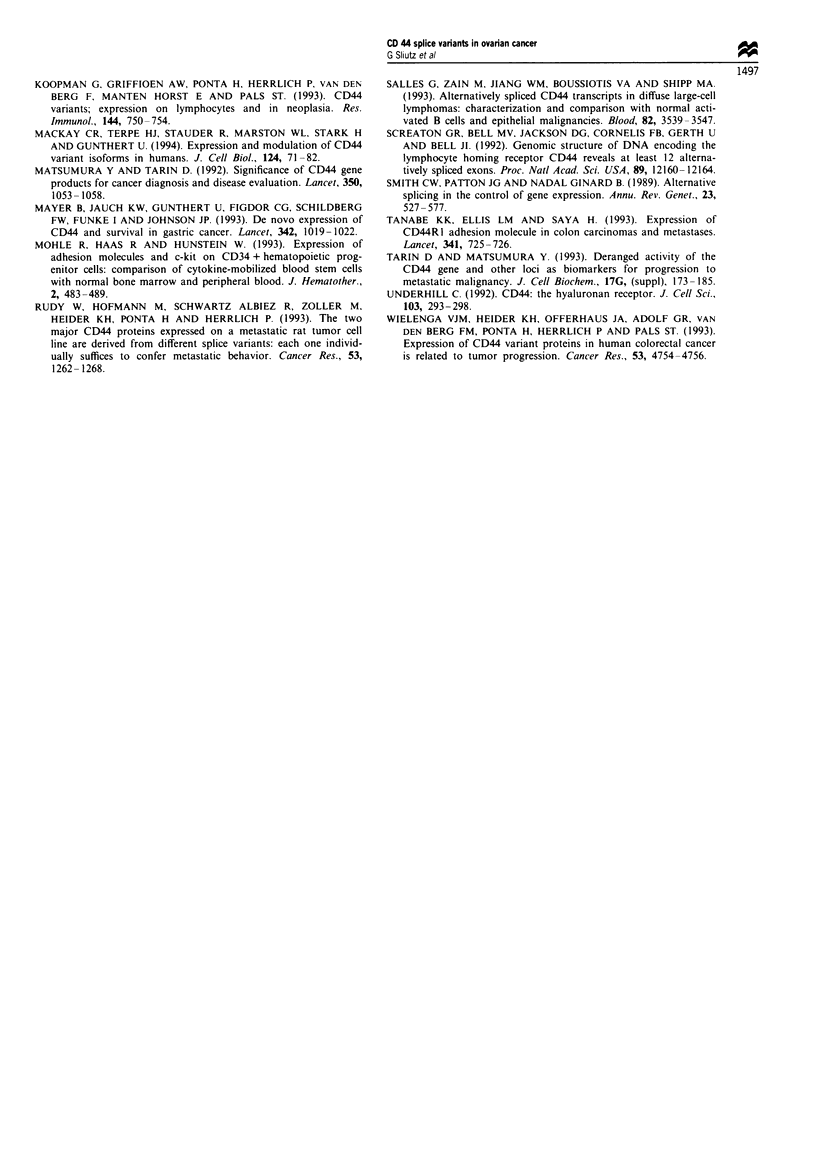# Immunohistochemical and serological evaluation of CD44 splice variants in human ovarian cancer.

**DOI:** 10.1038/bjc.1995.535

**Published:** 1995-12

**Authors:** G. Sliutz, C. Tempfer, S. Winkler, P. Kohlberger, A. Reinthaller, C. Kainz

**Affiliations:** Department of Gynaecology and Obstetrics, University of Vienna, Medical School, Austria.

## Abstract

The surface glycoprotein CD44 is widely distributed in different tissues. In contrast to healthy tissue, tumour samples show a more complex pattern of CD44 expression, indicating a loss of splice control. Beside cell-surface expression, the measurement of soluble CD44 in serum of cancer patients could be useful in early diagnosis and assessment of disease status. We evaluated the surface expression of CD44 isoforms in 22 ovarian cancer patients by means of immunohistochemistry. Additionally, we investigated 134 serological samples of these patients for the occurrence of CD44 isoform expression. For CD44 standard, CD44v5 and CF44v6 mean serum levels in patients with clinically detectable or non-detectable ovarian cancer were 422.4 +/- 143.8 ng ml-1 and 547.4 +/- 148.2 ng ml-1, 12.3 +/- 7.9 ng ml-1 and 21.9 +/- 12.2 ng ml-1 and 105.5 +/- 37.9 ng ml-1 and 144.9 +/- 50.9 ng ml-1 respectively (P-values not significant). CD44 surface proteins containing epitopes encoded by splice variants CD44v5, CD44v6 and CD44v7-8 were immunohistochemically detected in 9% (n = 2), 13% (n = 3) and 4% (n = 1) of the 22 tumour samples respectively. In the present study we showed that in ovarian cancer CD44 isoforms CD44v5 and CD44v6 are expressed in very low amounts by the tumours. In accordance with this, we found that the presence of tumour is not associated with higher serum levels of CD44standard, CD44v5 and CD44v6 in preoperative serum samples in ovarian cancer patients.


British Journal of Cancer (1995) 72, 1494–1497
© 1995 Stockton Press All rights reserved 0007–0920/95 $12.00

### Immunohistochemical and serological evaluation of CD44 splice variants in human ovarian cancer

G Sliutz[1], C Tempfer[1], S Winkler[2], P Kohlberger[1] A Reinthaller[1] and Ch Kainz[1]

*Departments of [1]Gynaecology and Obstetrics and [2]General Surgery, University of Vienna, Medical School, Vienna, Austria.*

**Summary** The surface glycoprotein CD44 is widely distributed in different tissues. In contrast to healthy tissue, tumour samples show a more complex pattern of CD44 expression, indicating a loss of splice control. Beside cell-surface expression, the measurement of soluble CD44 in serum of cancer patients could be useful in early diagnosis and assessment of disease status. We evaluated the surface expression of CD44 isoforms in 22 ovarian cancer patients by means of immunohistochemistry. Additionally, we investigated 134 serological samples of these patients for the occurrence of CD44 isoform expression. For CD44standard, CD44v5 and CF44v6 mean serum levels in patients with clinically detectable or non-detectable ovarian cancer were $422.4 \pm 143.8$ ng ml$^{-1}$ and $547.4 \pm 148.2$ ng ml$^{-1}$, $12.3 \pm 7.9$ ng ml$^{-1}$ and $21.9 \pm 12.2$ ng ml$^{-1}$ and 105.5 $\pm$ 37.9 ng ml$^{-1}$ and $144.9 \pm 50.9$ ng ml$^{-1}$ respectively (*P*-values not significant). CD44 surface proteins containing epitopes encoded by splice variants CD44v5, CD44v6 and CD44v7–8 were immunohistochemically detected in 9% ($n = 2$), 13% ($n = 3$) and 4% ($n = 1$) of the 22 tumour samples respectively. In the present study we showed that in ovarian cancer CD44 isoforms CD44v5 and CD44v6 are expressed in very low amounts by the tumours. In accordance with this, we found that the presence of tumour is not associated with higher serum levels of CD44standard, CD44v5 and CD44v6 in preoperative serum samples in ovarian cancer patients.

**Keywords** CD44; alternative splicing; ovarian neoplasms; serum; immunohistochemistry

Ovarian cancer is diagnosed at advanced stages in approximately 75% of patients. The prognosis in ovarian cancer remains poor, and there is a need to identify patients less likely to respond to treatment or patients who suffer from low-stage disease and have a worse prognosis. Early detection of ovarian cancer or of recurrent disease after primary therapy is very important in ovarian cancer patients. Serum tumour markers, which have been shown to be useful in early detection of primary tumours, recurrences, evaluation of prognosis and monitoring of therapy, have become an integral part of follow-up schemes in the management of ovarian cancer patients (Tarin and Matsumura, 1993; Dall *et al.*, 1994; Gold and Osband, 1994).

The surface glycoprotein CD44 is widely distributed in different tissues (Flanagan *et al.*, 1989). CD44 cell-surface adhesion molecules have been shown to mediate cell–cell and cell–matrix interactions (Screaton *et al.*, 1992), allowing circulating lymphocytes to migrate into lymphatic tissue (Jalkanen *et al.*, 1986; Salles *et al.*, 1993) and to control lymphocyte binding to peripheral lymph nodes, mucosal and synovial endothelial cells (Jalkanen *et al.*, 1987*a*). The CD44 family of transmembrane receptor molecules is derived from a single gene located on chromosome 11 (Koopman *et al.*, 1993). By the mechanism of 'messenger RNA alternative splicing' numerous isoforms of the CD44 glycoprotein are produced (Smith *et al.*, 1989; Matsumura and Tarin, 1992; Screaton *et al.*, 1992). Cell-surface proteins encoded by splice variants of CD44 differ from the CD44 standard isoform by additional amino acids in the extracellular region of the protein (Screaton *et al.*, 1992). CD44 isoforms encoded by splice variants occur on the surface of different normal cells (Mackay *et al.*; 1994). Tumour samples show a more complex pattern of CD44 expression, indicating a loss of splice control in malignantly transformed cells (Heider *et al.*, 1993). Rudy *et al.* (1993) could show that the expression of variants of the CD44 surface glycoprotein confers metastatic behaviour to a non-metastatic cell line in a rat carcinoma model (Gunthert *et al.*, 1991). In colorectal cancer, breast and cervical cancer as well as in gastrointestinal lymphoma and gastric cancer a correlation between the overexpression of specific isoforms of CD44 and poor prognosis was reported (Joensuu *et al.*, 1993*a*, *b*; Mayer *et al.*, 1993; Wielenga *et al.*, 1993; Kainz *et al.*, 1995*a*).

It was speculated that the overexpression of specific CD44 isoforms, mainly CD44 splice variants v5, v6 and v7–8, could raise the metastatic potential of a tumour by facilitating the expansion of malignant cells into the draining lymph nodes (Salles *et al.*, 1993).

Antibodies raised against different soluble CD44 molecules allow the detection of CD44 isoforms in serum samples. The measurement of soluble CD44 molecules in body fluids could be useful in early diagnosis of cancer, assessment of disease status and evaluation of metastatic potential and prognosis.

In a recent study we investigated the serum levels of proteins encoded by CD44 splice variants in cervical cancer patients and found significantly elevated levels of CD44v6 when tumour was present (Kainz *et al.*, 1995*b*). AG Zeimet *et al.* (personal communication) describe high serum levels of the CD44standard molecule in ovarian cancer patients when tumour is present.

This prompted us to evaluate the levels of CD44 glycoproteins encoded by CD44standard and CD44 splice variants v5 and v6 in serum samples of women suffering from ovarian cancer. Furthermore, we evaluated the immunohistochemical expression of epitopes encoded by CD44 splice variants v5, v6 and v7–8 in tumour specimens from the same sample of patients.

### Patients and methods

This study includes 134 clinical and serological examinations of 22 patients suffering from ovarian cancer FIGO stages I–IV. All patients were treated by hysterectomy, lymph node sampling and omentectomy followed by a platinum-containing chemotherapy. All patients underwent a close follow-up programme consisting of regular visits at 3 month intervals, including vaginorectal palpation and ultrasound examination of the lower abdominal tract. Computertomography of the pelvis was performed every 6 months. Serum samples were taken before therapy and during a follow-up period of at least 12 months and up to 36 months and were stored at −20°C.

Correspondence: G Sliutz, Department of Gynaecology and Obstetrics, University of Vienna, Spitalgasse 23, A-1090 Vienna, Austria.
Received 27 March 1995; revised 17 July 1995; accepted 17 July 1995

The patients were selected randomly. One preoperative serum sample and a minimum of four post-operative serum samples were available from every patient. In cases of tumour relapse at least five post-operative serum samples before the time of clinical evidence of relapse were available.

*Serum assay*

Serum levels of soluble CD44 isoforms CD44standard, CD44v5 and CD44v6 were measured using the sCD44standard (st) enzyme-linked immunosorbent assay (ELISA), sCD44var (v5) ELISA and sCD44var (v6) ELISA (Bender Med Systems, Bender, Vienna, Austria) respectively. All tests were run in duplicate according to manufacturer's instructions and without knowledge of the clinical outcome. A panel of 22 sera from healthy blood donors were tested for serum levels of CD44standard, CD44v5 and CD44v6.

To define the specificity of these ELISAs several structurally related and non-related polypeptides were tested for cross-reactivity. There is no detectable cross-reactivity with CD44 polypeptides lacking the protein sequence encoded by the corresponding exons. The limit of detection of soluble CD44 molecules (sensitivity) by antibodies used in these experiments was determined to be between 0.07 and 0.22 ng $ml^{-1}$. Intra-assay and inter-assay reproducibility has been calculated to be between 3% and 5.8%.

*Immunohistochemistry*

A total of 22 routinely formalin-fixed and paraffin-embedded surgery samples were used to evaluate the expression of epitopes encoded by CD44 splice variants CD44v5, CD44v6 and CD44v7-8. The paraffin sections were soaked in xylene to remove paraffin and rehydrated in a graded alcohol series (100-70%). To recover antigenicity we used the 'Antigen Retrieval System' (Bio Genex, San Ramon, CA, USA) twice for 20 min in a microwave at 600 W power (HM 146, Elektra Bregenz, Schwaz, Austria) and then the sections were washed in 10 mM phosphate-buffered saline (PBS) (pH 7.6). Three different primary antibodies to different epitopes encoded by CD44 splice variants were used. The first was a monoclonal antibody specific for the epitope encoded by exon v5 of human variant CD44 (CD44v5, clone VFF-8, Bender), the second monoclonal antibody was specific for the epitope encoded by exon v6 of human variant CD44 (CD44v6, clone VFF-7, Bender) and the third monoclonal antibody was specific for the epitope encoded by exons v7-8 of human variant CD44 (CD44v7-8, clone VFF-17, Bender). The primary antibody was diluted in serum/PBS and the sections were incubated for 60 min and then incubated for 30 min with biotinylated anti-mouse and anti-rabbit link antibody (Dako LSAB 2 Kit, Dako, Carpinteria, CA, USA). After rinsing in PBS the sections were coated with streptavidin conjugated to alkaline phosphatase for 10 min. The sections were then rinsed in PBS, incubated with fast red chromogen (naphtol phosphate substrate in Tris buffer, fast red chromogen tablets 5 mg, Bio Genex, San Ramon, CA, USA) and then washed with distilled water. The sections were finally counterstained with haematoxylin and mounted. We interpreted strong and/or widespread staining as positive, weak and focal staining as negative.

*Positive control* The positive control slide was prepared from epidermal tissue known to contain the antigen. In the positive control tissue all monoclonal antibodies stained similarly.

*Negative control* The negative control slide was prepared from the same tissue block as the specimen. Instead of the primary antibody we used a normal, non-immune serum supernatant from the same source as the primary antibody.

*Statistics*

All results are expressed as mean ± standard error of the mean. Depending on the type and distribution of the data group, comparisons were achieved by analysis of variance (ANOVA) procedures or by Wilcoxon two-sample test. The significance level assumed was $P = 0.05$.

### Results

Mean age of the patients at the time of diagnosis was 57.6 ± 16.7 (range 26-89) years. Ovarian cancer stages I, II, III and IV were present in three, five, ten and four cases. Serous, mucinous cystadenocarcinoma, undifferentiated carcinoma and adenocarcinoma were present in nine, eight, three and two cases respectively. Our material included 76 and 58 serum samples of women with and without evidence of disease respectively.

The detected mean levels of healthy donors were 443 ± 125 ng $ml^{-1}$, 35 ± 13 ng $ml^{-1}$ and 170 ± 54 ng $ml^{-1}$ for CD44standard, CD44v5 and CD44v6 respectively. For CD44standard mean serum levels in patients with clinically detectable or clinically non-detectable ovarian cancer were 422.4 ± 143.8 ng $ml^{-1}$ and 547.4 ± 148.2 ng $ml^{-1}$ respectively (*P*-value not significant). For CD44v5 we measured a mean serum concentration in serum samples with clinically detectable tumour or not of 12.3 ± 7.9 ng $ml^{-1}$ and 21.9 ± 12.2 ng $ml^{-1}$ respectively (*P*-value not significant). Proteins encoded by the splice variant CD44v6 showed a mean concentration of 105.5 ± 37.9 ng $ml^{-1}$ when tumour was present and a mean concentration of 144.9 ± 50.9 ng $ml^{-1}$ in cases of complete remission (*P*-value not significant).

Serum levels of CD44 isoforms CD44standard, CD44v5 CD44v6 and CD44v7, grouped by the following clinical features: before therapy, complete remission, partial remission, steady disease and progression, are shown in Table I.

We found no significant differences between mean serum levels when pre-treatment samples were grouped by stage, histological type of tumour and lymph node involvement. Eight patients developed recurrent disease after complete remission. No increase of serum levels of CD44st, CD44v5 and CD44v6 could be observed before clinical evidence of relapse.

*Immunohistochemistry*

CD44 surface proteins containing epitopes encoded by splice variants CD44v5, CD44v6 and CD44v7-8 were detected by means of immunohistochemistry in two, three and one case of the 22 tumour samples respectively. We found homogeneous staining in tumours that were considered positive for CD44 expression.

We found no correlation between expression of any of the splice variants and histological type, stage of the tumour or age of the patient.

**Table I** Mean serum levels of CD44standard, CD44v5, CD44v6 and CD44v7 grouped by clinical status expressed as mean ± s.d.

| | *Before therapy* | *Complete remission* | *Partial remission* | *Steady disease* | *Progression* |
|---|---|---|---|---|---|
| CD44standard (ng $ml^{-1}$) | 468.2 ± 137.6 | 547.4 ± 148.2 | 359.1 ± 106.2 | 371.3 ± 132.9 | 548.8 ± 142.1 |
| CD44v5 (ng $ml^{-1}$) | 14.6 ± 12.2 | 22.0 ± 12.2 | 12.3 ± 4.0 | 12.6 ± 5.8 | 9.5 ± 9.5 |
| CD44v6 (ng $ml^{-1}$) | 111.2 ± 46.8 | 144.9 ± 50.9 | 110.1 ± 32.0 | 99.9 ± 27.3 | 93.7 ± 46.5 |
| CD44v7 (ng $ml^{-1}$) | 2.5 ± 3.2 | 1.6 ± 3.1 | 2.3 ± 3.7 | 0.2 ± 0.6 | 0.3 ± 0.7 |

Mean serum levels of CD44standard, CD44v5, CD44v6 and CD44v7 grouped by clinical status expressed as mean ± s.d.

### Discussion

In a previous study we were able to show that in cervical cancer patients' elevated CD44v6 serum levels were significantly correlated with clinical evidence of disease (Kainz *et al.*, 1995*b*). CD44 isoforms CD44v5 and CD44v7–8 were strongly expressed by the tumour cells in cervical cancer patients. The expression of the CD44 isoform CD44v6 on tumour cells has been reported to be an independent prognostic factor in surgically treated cervical cancer patients (Kainz *et al.*, 1995*c*). Although the two abovementioned studies examined different patient collectives, the available data lead to the conclusion that the expression of CD44v6 on tumour cells is reflected in its serum level.

In the present study we show that in this ovarian cancer collective the immunohistochemically detected expression rate of the CD44 isoforms CD44v5 and CD44v6 is very low. Consequently, serum levels of CD44v5 and CD44v6 in preoperative samples are not elevated compared with healthy controls.

Aberrant expression of glycoproteins encoded by CD44 splice variants has been detected by means of immunohistochemistry in human paraffin-embedded tumour samples (Heider *et al.*, 1993; Tanabe *et al.*, 1993). The expression of specific CD44 isoforms has been shown to be associated with metastasis and to be of prognostic relevance in human malignancies such as colorectal cancer, breast and cervical cancer, gastrointestinal lymphoma and gastric cancer (Joensuu *et al.*, 1993*a*; Kainz *et al.*, 1995*a*; Mayer *et al.*, 1993; Wielenga *et al.*, 1993).

Our data show that in ovarian cancer serum levels of the CD44 isoforms CD44v5 and CD44v6 do not reflect the tumour burden. Confirming that finding, we were able to show, by means of immunohistochemistry, that epitopes encoded by CD44 splice variants CD44v5, CD44v6 and CD44v7–8 are detectable in very low amounts in paraffin-embedded tissue samples of ovarian cancer patients. No correlation with any clinical or histological parameter was found.

When grouped by clinical status the highest serum levels of the CD44standard isoform were found in cases of tumour progression and in cases of complete remission. This is surprising since pretherapeutic serum levels of CD44standard are not significantly elevated and thus serum levels of CD44standard does not seem to correlate with the presence of tumour.

All patients in our collective underwent a post-operative chemotherapy with six cycles of carboplatin (400 mg $m^{-2}$) and cyclophosphamide (600 mg $m^{-2}$) in a 4 week interval. In the cases of tumour relapse or progression second-line chemotherapy with paclitaxel (17 mg $m^{-2}$) was applied after a 3 week interval. In order to stimulate peripheral blood progenitor cells patients were given granulocyte–macrophage colony-stimulating factor (GM-CSF; 5 μg $kg^{-1}$) (de Vries *et al.*, 1991).

The CD44standard isoform is distributed on haematopoietic cells, including all subsets of leucocytes, monocytes and erythrocytes and on different non-haematopoietic cells (Jalkanen *et al.*, 1987*b*; Flanagan *et al.*, 1989; Underhill, 1992). High-dose chemotherapy with the support of peripheral blood progenitor cells (PBPCs) is increasingly used in the treatment of solid tumours. Inducing PBPC proliferation by colony-stimulating factors results in up-regulation of CD34 expression. These cells do also express the CD44-standard isoform in almost 95% (Mohle *et al.*, 1993; Dicke *et al.*, 1994).

An increase of haematopoietic cells expressing the CD44-standard isoform could be responsible for the elevated CD44st serum levels.

We conclude that CD44 isoforms CD44standard, CD44v6 and CD44v7–8 are expressed at a very low level in malignant ovarian tumours. Serum levels of soluble CD44 isoforms do not reflect the tumour burden. Serum CD44standard showed higher levels when high haematopoietic activity was present. Therefore we hypothesise that the serum CD44standard production in haematopoietic cells overrules the production of ovarian tumour cells. The serum level of CD44standard mostly reflects the haematopoietic activity in these patients.

To clarify this matter we are currently examining the influence of growth factors used in chemotherapy regimens on the expression of CD44 isoforms.

**Acknowledgements**
This work was supported by a grant from the mayor of Vienna to Gerhard Sliutz.

### References

DALL P, HEIDER KH, HEKELE A, VON MINCKWITZ G, KAUFMANN M, PONTA H AND HERRLICH P. (1994). Surface protein expression and messenger RNA-splicing analysis of CD44 in uterine cervical cancer and normal cervical epithelium. *Cancer Res.*, **54**, 3337–3341.

DE VRIES EG, BIESMA B, WILLEMSE PH, MULDER NH, STERN AC, AALDERS JG AND VELLENGA E. (1991). A double-blind placebo-controlled study with granulocyte-macrophage colony-stimulating factor during chemotherapy for ovarian carcinoma. *Cancer Res.*, **51**, 116–122.

DICKE KA, HOOD D AND HANKS S. (1994). Peripheral blood stem cell collection after mobilization with intensive chemotherapy and growth factors. *J. Hematother.*, **3**, 141–144.

FLANAGAN BF, DALCHAU R, ALLEN AK, DAAR AS AND FABRE JW. (1989). Chemical composition and tissue distribution of the human CDw44 glycoprotein. *Immunology*, **67**, 167–175.

GOLD JE AND OSBAND ME. (1994). Autolymphocyte therapy. II. Dependence of in vivo anti-tumour specificity and long-term immunity against murine melanoma and carcinoma on ex vivo activated donor memory T-cells. *Clin. Immunol. Immunopathol.*, **71**, 325–332.

GUNTHERT U, HOFMANN M, RUDY W, REBER S, ZOLLER M, HAUSSMANN I, MATZKU S, WENZEL A, PONTA H AND HERRLICH P. (1991). A new variant of glycoprotein CD44 confers metastatic potential to rat carcinoma cells. *Cell*, **65**, 13–24.

HEIDER KH, DAMMRICH J, SKROCH ANGEL P, MULLER HERMELINK HK, VOLLMERS HP, HERRLICH P AND PONTA H. (1993). Differential expression of CD44 splice variants in intestinal and diffuse-type human gastric carcinomas and normal gastric mucosa. *Cancer Res.*, **53**, 4197–4203.

JALKANEN S, STEERE AC, FOX RI AND BUTCHER EC. (1986). A distinct endothelial cell recognition system that controls lymphocyte traffic into inflamed synovium. *Science*, **233**, 556–558.

JALKANEN S, BARGATZE RF, DE LOS TOYOS J AND BUTCHER EC. (1987*a*). Lymphocyte recognition of high endothelium: antibodies to distinct epitopes of an 85–95kD glycoprotein antigen differentially inhibit lymphocyte binding to lymph node, mucosal, or synovial endothelial cells. *J. Cell Biol.*, **105**, 983–990.

JALKANEN S, WU N, BARGATZE RF AND BUTCHER EC. (1987*b*). Human lymphocyte and lymphoma homing receptors. *Annu. Rev. Med.*, **38**, 467–476.

JOENSUU H, KLEMI PJ, TOIKKANEN S AND JALKANEN S. (1993*a*). Glycoprotein CD44 expression and its association with survival in breast cancer. *Am. J. Pathol.*, **143**, 867–874.

JOENSUU H, RISTAMAKI R, KLEMI PJ AND JALKANAN S. (1993*b*). Lymphocyte homing receptor (CD44) expression is associated with poor prognosis in gastrointestinal lymphoma. *Br. J. Cancer*, **68**, 428–432.

KAINZ C, KOHLBERGER P, TEMPFER C, SLUITZ G, REINTHALLER A AND BREITENECKER G. (1995*a*). Prognostic value of CD44 splice variants in human cervical cancer stage III. *Eur. J. Cancer*. (In Press).

KAINZ C, TEMPFER C, WINKLER S, SLIUTZ G, KOELBL H AND REINTHALLER A. (1995*b*). Serum CD44 splice variants in cervical cancer patients. *Cancer Lett.*, **90**, 231–234.

KAINZ C, KOHLBERGER P, SLIUTZ G, TEMPFER C, HEINZL H, REINTHALLER A, BREITENECKER G AND KOELBL H. (1995*c*). Splice variants of CD44 in human cervical cancer stage IB to IIB. *Gynecol. Oncol.* (In Press).

KOOPMAN G, GRIFFIOEN AW, PONTA H, HERRLICH P, VAN DEN BERG F, MANTEN HORST E AND PALS ST. (1993). CD44 variants; expression on lymphocytes and in neoplasia. *Res. Immunol.*, **144,** 750–754.

MACKAY CR, TERPE HJ, STAUDER R, MARSTON WL, STARK H AND GUNTHERT U. (1994). Expression and modulation of CD44 variant isoforms in humans. *J. Cell Biol.*, **124,** 71–82.

MATSUMURA Y AND TARIN D. (1992). Significance of CD44 gene products for cancer diagnosis and disease evaluation. *Lancet*, **350,** 1053–1058.

MAYER B, JAUCH KW, GUNTHERT U, FIGDOR CG, SCHILDBERG FW, FUNKE I AND JOHNSON JP. (1993). De novo expression of CD44 and survival in gastric cancer. *Lancet*, **342,** 1019–1022.

MOHLE R, HAAS R AND HUNSTEIN W. (1993). Expression of adhesion molecules and c-kit on CD34 + hematopoietic progenitor cells: comparison of cytokine-mobilized blood stem cells with normal bone marrow and peripheral blood. *J. Hematother.*, **2,** 483–489.

RUDY W, HOFMANN M, SCHWARTZ ALBIEZ R, ZOLLER M, HEIDER KH, PONTA H AND HERRLICH P. (1993). The two major CD44 proteins expressed on a metastatic rat tumor cell line are derived from different splice variants: each one individually suffices to confer metastatic behavior. *Cancer Res.*, **53,** 1262–1268.

SALLES G, ZAIN M, JIANG WM, BOUSSIOTIS VA AND SHIPP MA. (1993). Alternatively spliced CD44 transcripts in diffuse large-cell lymphomas: characterization and comparison with normal activated B cells and epithelial malignancies. *Blood*, **82,** 3539–3547.

SCREATON GR, BELL MV, JACKSON DG, CORNELIS FB, GERTH U AND BELL JI. (1992). Genomic structure of DNA encoding the lymphocyte homing receptor CD44 reveals at least 12 alternatively spliced exons. *Proc. Natl Acad. Sci. USA*, **89,** 12160–12164.

SMITH CW, PATTON JG AND NADAL GINARD B. (1989). Alternative splicing in the control of gene expression. *Annu. Rev. Genet.*, **23,** 527–577.

TANABE KK, ELLIS LM AND SAYA H. (1993). Expression of CD44R1 adhesion molecule in colon carcinomas and metastases. *Lancet*, **341,** 725–726.

TARIN D AND MATSUMURA Y. (1993). Deranged activity of the CD44 gene and other loci as biomarkers for progression to metastatic malignancy. *J. Cell Biochem.*, **17G,** (suppl), 173–185.

UNDERHILL C. (1992). CD44: the hyaluronan receptor. *J. Cell Sci.*, **103,** 293–298.

WIELENGA VJM, HEIDER KH, OFFERHAUS JA, ADOLF GR, VAN DEN BERG FM, PONTA H, HERRLICH P AND PALS ST. (1993). Expression of CD44 variant proteins in human colorectal cancer is related to tumor progression. *Cancer Res.*, **53,** 4754–4756.